# Mechanistic Studies of Lactate in Heart Failure: From Metabolic Biomarker to Therapeutic Target

**DOI:** 10.31083/RCM47433

**Published:** 2026-07-14

**Authors:** Zhongjian Zhang, Ting Zeng, Xiaoyu Li, Zhiling He, Shuang Li

**Affiliations:** ^1^Department of Cardiology, General Hospital of Western Theater Command, 610083 Chengdu, Sichuan, China

**Keywords:** lactate, heart failure, metabolic biomarker, therapeutic target, myocardial remodeling, inflammatory response

## Abstract

Heart failure (HF) is a significant cardiovascular syndrome with a high prevalence of morbidity and mortality across the globe. Meanwhile, lactate, a byproduct of glycolysis, has been implicated in myocardial hypoxia and disarranged energy metabolism and has drawn attention in the context of HF. In addition to serving as a highly sensitive biomarker, lactate might also contribute to myocardial remodeling and inflammation via multiple signaling pathways, thereby affecting disease course and prognosis; however, the mechanisms through which lactate impacts HF remain incompletely elucidated, and published results are contradictory. Therefore, this review encompassed lactate metabolism and the associated physiological and pathological functions in HF, underlining the clinical significance of lactate as a metabolic biomarker. Moreover, this review discussed recent progress targeting lactate metabolism or lactylation as potential treatments to promote a clearer understanding of the mechanistic aspects of metabolic regulation in HF and to guide clinical diagnosis and treatment.

## 1. Introduction

Heart failure (HF) remains a global health burden with high morbidity and mortality, its prevalence fueled by aging populations and comorbidities such as diabetes. Despite therapeutic advances, HF prognosis is variable and management is complicated by diverse phenotypes, underscoring the persistent need to elucidate underlying mechanisms and identify novel therapeutic targets [1].

There has been a fundamental alteration in how lactate is perceived; it was once regarded simply as a by-product of anaerobic glycolysis but now it is acknowledged as an essential energy source, a signaling substance, and a controller of immune and cellular operations and this “lactate transformation” is best illustrated by the finding of histone lactylation which is an epigenetic adjustment that directly connects lactate metabolism to gene regulation and in cardiovascular disorders, the dynamics of lactate significantly impact myocardial energy, vascular performance, inflammation, and remolding underlining its potential as a vital factor in disease processes [2,3].

This review presents a targeted integration of lactate’s functions in heart failure (HF), delving into three main areas: firstly, the mechanisms by which lactate and lactylation are involved in the remolding and malfunction of myocardial metabolism, secondly, the significance of lactate as a dynamic biomarker for diagnosis and prognosis in both acute heart failure (AHF) and chronic HF, and thirdly, the possibilities and constraints of targeting lactate metabolism or lactylation for treatment purposes, and through the combination of these elements, the aim was to elucidate how lactate connects biomarker-related biological processes to therapeutic chances in HF.

## 2. Main Body

### 2.1 Metabolic Characteristics of Lactate

#### 2.1.1 Mechanisms of Lactate Production and Clearance

Lactate is much more than a simple metabolic byproduct, it is now recognized as a central metabolite and crucial signaling molecule with complex roles in cellular energy production and adaptation. Its synthesis is catalyzed by lactate dehydrogenase (LDH), which reversibly converts pyruvate to lactate under both anaerobic and aerobic conditions—the latter underpinning the physiologically crucial “lactate shuttle” [4]. The LDH isozymes, lactate dehydrogenase A (*LDHA*; favoring lactate generation) and lactate dehydrogenase B (*LDHB*; promoting lactate oxidation), are differentially regulated: during moderate exercise, enhanced lactate flux supports energy transfer between tissues, a capacity elevated in endurance-trained individuals [5]. In the heart, lactate serves as a vital oxidative fuel for cardiomyocytes, especially during increased workload [6,7].

Lactate transfer is regulated by monocarboxylate transporters (MCTs), with monocarboxylate transporter 1 (MCT1) promoting lactate intake into oxidative tissues such as the heart for mitochondrial oxidation while monocarboxylate transporter 4 (MCT4) expels lactate from glycolytic cells, and genetic research has demonstrated that a lack of MCT1 alters muscle metabolism by increasing nicotinamide adenine dinucleotide (NAD⁺) levels, activating the sirtuin 1 (SIRT1)/peroxisome proliferator-activated receptor (PPAR) gamma coactivator 1 alpha (PGC-1α) signaling pathway, and strengthening mitochondrial formation and the tricarboxylic acid (TCA) cycle flow [8,9], and the mitochondrial pyruvate carrier (MPC) further modulates this system; when it malfunctions, it hinders pyruvate’s entry into mitochondria, resulting in lactate buildup and worsening abnormal cardiac remodeling [10].

In situations like heart failure (HF), sepsis, or cardiogenic shock where things go wrong physiologically, lactate builds up because the body can’t get rid of it properly and there’s more anaerobic glycolysis happening, this messes up the redox balance makes metabolism less flexible and is linked to bad outcomes [11,12,13,14,15], continual high levels of lactate indicate not just a lack of oxygen but also uncontrolled inflammatory and fibrotic reactions [16,17], lactate acts as a signaling molecule through the receptor called G protein-coupled receptor 81 (GPR81) which is also named hydroxycarboxylic acid receptor 1 (HCAR1) and has an impact on lipolysis and glucose homeostasis in an autocrine and paracrine manner [18], additionally, lactate causes lysine lactylation (Kla), a new type of post-translational modification, on histones and metabolic enzymes directly connecting lactate amounts to epigenetic changes and different enzyme activities in HF and immune system activation [19].

**Fig. 1.**
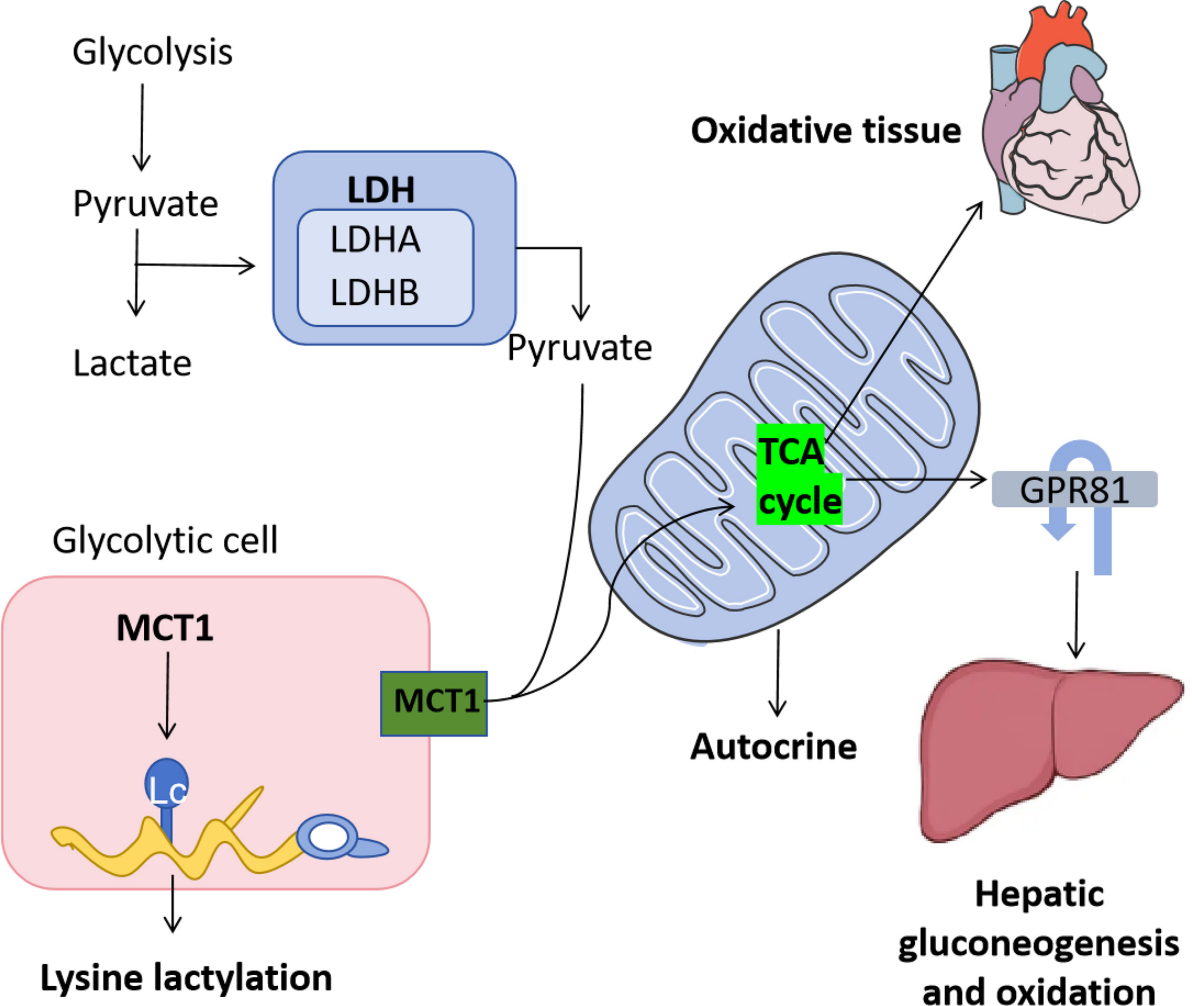
**Mechanisms of lactate production and clearance**. LDH, lactate dehydrogenase; LDHA, lactate dehydrogenase A; LDHB, lactate dehydrogenase B; MCT1, monocarboxylate transporter 1; TCA, tricarboxylic acid; GPR81, G protein-coupled receptor 81. Blue meandering arrow: lactate (or its metabolic signal) activates GPR81 in an autocrine manner.

Hepatic lactate clearance (LC) plays a crucial part in maintaining systemic homeostasis mainly via gluconeogenesis and oxidation and is controlled by enzymes such as LDH and the bile acid receptor farnesoid X receptor [20,21], the complex regulation of lactate generation, transportation, and clearance highlights its two-fold function as both a metabolic intermediate and a signal that modifies diseases, so targeting its synthesis (for instance, *LDHA* inhibitors), transportation (MCT modulators), or signaling pathways in therapy has great potential for treating metabolic and inflammatory diseases like heart failure (HF) (Results are shown in Fig. 1).

#### 2.1.2 Lactate as an Energy Substrate Utilization

Lactate can be oxidized in the myocardium after conversion to pyruvate, which enters the tricarboxylic acid (TCA) cycle to support oxidative phosphorylation and ATP production. Recent work suggests that lactate may also be transported into mitochondria via monocarboxylate transporter 1 (MCT1), bypassing the mitochondrial pyruvate carrier (MPC) and supporting respiration by generating NADH, even when TCA flux is impaired. This pathway may help maintain cardiac energetics under stress such as ischemia or HF, where mitochondrial function is compromised [22]. Human studies using hyperpolarized 13C-pyruvate magnetic resonance spectroscopy further show that myocardial pyruvate-to-lactate conversion is dynamic and tracks metabolic demand and glycemic state, highlighting lactate’s flexibility as a substrate across physiological conditions [23].

In pathological states like diabetic cardiomyopathy, lactate metabolism gets modified which was shown by elevated myocardial lactate levels signifying a shift towards more glycolysis and less oxidative metabolism; this alteration made energy less efficient and increased vulnerability to low-oxygen situations and interventions that changed lactate metabolism, for example carnitine supplementation and adjusting lactate transporters, had potential in improving myocardial energy and function in experimental models; also the build-up of lactate and its metabolic consequences were linked to myocardial ischemia-reperfusion injury in which too much lactate and reactive oxygen species worsened mitochondrial problems and led to cell death and treatments aimed at making lactate metabolism normal again showed heart-protective effects, like stopping LDH and changing lactylation, a post-translational modification.

#### 2.1.3 Lactate and Metabolic Remodeling in Cardiomyocytes

Lactate serves as a key metabolic substrate and a potent signaling molecule that drives metabolic remodeling in cardiomyocytes, especially during abnormal states like thickening of the heart muscle (hypertrophy) and heart failure (HF), and this alteration from burning fat-derived acids for energy to sugar-based metabolism entails increasing the levels of LDH, MCTs, and MPCs, for example, in diabetic hearts, too much MCT4 causes excessive lactate to leave the cells, upsetting the regular balance between lactate and pyruvate, leading to more oxidative stress and inflammation, worsening heart muscle damage [24], on the molecular scale, lactate-induced lactylation, a new kind of post-translational adjustment, changes both histone and non-histone proteins, reforming energy metabolic enzymes and inflammatory routes when under stress [25], also, lactate triggers signaling chains, such as hypoxia inducible factor 1 alpha (HIF-1α) and Janus kinase/signal transducer and activator of transcription, which further boost glycolytic activity and inflammatory reactions, promoting fibrosis and improper heart remodeling [26,27].

Besides cell-autonomous mechanisms, lactate promotes intercellular metabolic communication within the heart and the lactate transfer from cardiac fibroblasts to cardiomyocytes was helpful in maintaining the energy supply during hypertensive stress, emphasizing lactate’s function in coordinating tissue-level metabolic adjustment [28], and these mechanisms underlined the possibility of targeting lactate transportation and metabolism to regulate remodeling procedures and enhance cardiac function in heart failure (HF).

To sum up, lactate is conducive to myocardial energetics via mitochondrial oxidation and also functions as an epigenetic and signaling modulator which has an impact on gene expression, metabolic adaptability, and inflammatory reactions; a more profound comprehension of the underlying mechanisms regarding these two-fold functions—particularly the regulation mediated by lactylation and the intercellular transportation of lactate—might pave the way for novel therapeutic approaches to treat HF and ischemic heart disease by zeroing in on lactate-associated pathways.

#### 2.1.4 Regulation of Lactylation: Writers and Erasers

**Fig. 2.**
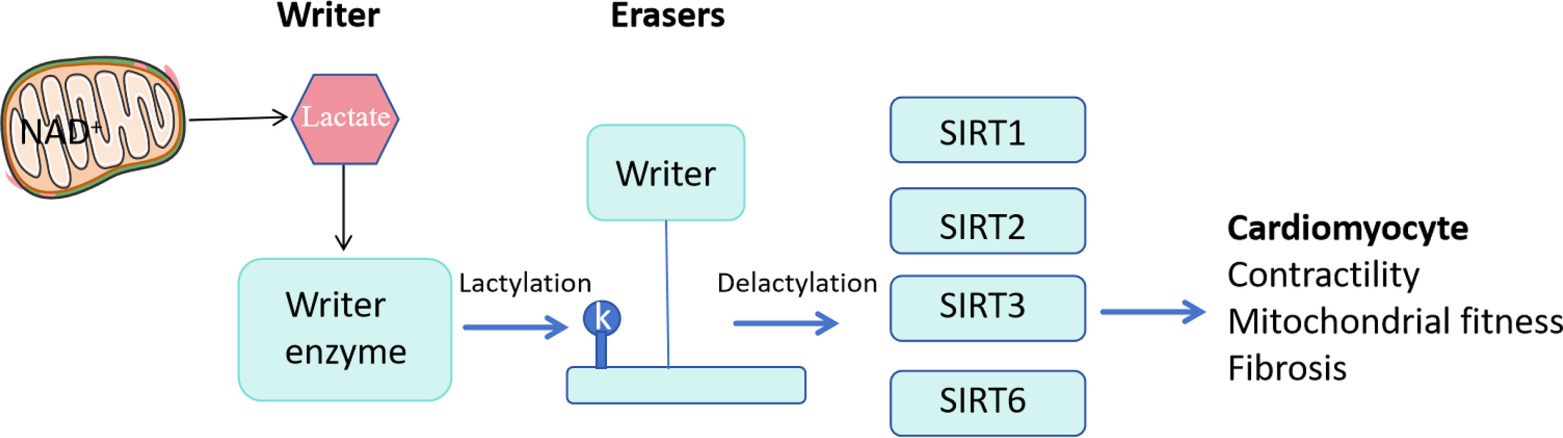
**Regulation of lactylation: writers and erasers**. NAD^+^, nicotinamide adenine dinucleotide; SIRT1, sirtuin 1; SIRT2, sirtuin 2; SIRT3, sirtuin 3; SIRT6, sirtuin 6.

A growing amount of evidence indicates that a number of sirtuins (SIRTs), like sirtuin 1 (SIRT1), sirtuin 2 (SIRT2), sirtuin 3 (SIRT3), and sirtuin 6 (SIRT6), have the ability to remove lysine lactylation and can take away histone lactyl modifications both outside and inside cells [29,30,31], which makes lactylation a dynamic and reversible post-translational modification similar to other acylations and it is regulated by special enzymatic “erasers” whose functioning might rely on metabolic situations, the availability of NAD^+^, and interactions with other modifications; however, the functional importance of lactylation—especially in heart failure—has not been fully explored yet and up to now, most of the understanding about its mechanisms comes from cancer or immune cells in which lactylation affects gene expression, growth, and metabolism [30,32] but there is no direct proof showing cause and effect in cardiomyocytes or cardiac fibroblasts connecting specific lactylation sites to functional results such as how well the heart contracts, the healthiness of mitochondria, or the development of fibrosis so future research should give priority to* in vivo* models, for example, introducing mutations at crucial lactylation sites and manipulating specific cell types along with thoroughly examining the structure and function of the heart to figure out the pathophysiological functions of lactylation in the heart (Results are shown in Fig. 2).

### 2.2 Lactate as a Metabolic Marker for HF

#### 2.2.1 Correlation Between Lactate Levels and HF

##### 2.2.1.1 Clinical Monitoring of Lactate Levels and Prognostic Assessment

Lactate plays a crucial part in the clinical monitoring and prognosis evaluation of heart failure (HF), as it mirrors the metabolic and hemodynamic states of patients; high lactate levels indicate tissue under-perfusion and anaerobic metabolism, which are often seen in acute heart failure (AHF) and chronic HF; research has shown that the level of lactate in arterial blood serves as an independent predictor of death from any cause during hospitalization for patients with AHF admitted to intensive care units and higher lactate quartiles are connected to a greater risk of death; when lactate measurements are combined with existing severity assessment methods, like the Simplified Acute Physiology Score II, the accuracy of prognosis improves and the precision of risk classification is enhanced; moreover, lactate levels have a positive association with N-terminal pro-B-type natriuretic peptide, a well-known biomarker showing cardiac stress, implying a pathophysiological link between metabolic disorders and cardiac malfunction; in chronic HF, high lactate levels are also related to poor outcomes, including higher death and rehospitalization rates; for instance, the lactate/albumin ratio (LAR) is a better prognostic indicator than lactate or albumin separately, as it can predict both short-term and long-term death in critically ill patients with HF; additionally, plasma lactate concentrations are associated with the severity of myocardial fibrosis and cardiac dysfunction in patients with dilated cardiomyopathy and HF, stressing the role of lactate as a manifestation of underlying myocardial remolding processes; clinically, lactate is applicable to risk prediction systems, such as the Get With The Guidelines-Heart Failure score, and adding lactate measurements strengthens the predictive power for in-hospital death; overall, these findings underline the importance of regular lactate monitoring in HF patients for directing prognosis assessments and treatment choices.

##### 2.2.1.2 Application of Dynamic Lactate Monitoring in AHF and Chronic HF

The dynamic evaluation of lactate concentrations provides crucial understandings regarding the varying metabolic states and therapeutic reactions in both acute heart failure (AHF) and chronic heart failure (CHF) situations, and unlike static assessments, repeated lactate tests along with related measures such as lactate clearance (LC) offer more comprehensive prognostic details, also high lactate levels in patients with cardiogenic shock (CS), a serious manifestation of heart failure, are highly associated with a greater risk of death, and reaching early hemodynamic goals, like achieving a cardiac power output index of no less than 0.30 W/m^2^ following Impella assistance, is connected to better LC and higher survival rates [33], additionally, lactate measurements weighted by time over a 24-hour span, which combine lactate figures over time to show overall exposure, have been independently related to negative in-hospital results among AHF patients, stressing the significance of including temporal lactate alterations in clinical assessments [34], on the other hand, in chronic HF models, lactate levels measured during exercise have been utilized to objectively measure fatigue and physiological adjustment, and blood lactate concentrations at the point of exhaustion act as a dependable marker of exercise capacity and training response [35], moreover, non-invasive methods, such as sweat lactate sensors, have shown themselves to be both secure and precise in detecting anaerobic thresholds in HF patients, possibly allowing for real-time surveillance in clinical settings [36], it should be noted that normal lactate levels don’t exclude hemodynamic instability, as research has shown differences between lactate levels and invasive hemodynamic indicators like cardiac index, underlining the need for a complete evaluation that goes beyond just lactate measurements [37], these effects are brought about by mechanisms involving the disruption of vascular endothelial-cadherin and GPR81 signaling, and such pathways might impact disease development and present potential targets for treatment [38], in short, the constant monitoring of lactate levels together with clinical and hemodynamic data enhances prognostic accuracy and guides individualized management plans for HF patients.

#### 2.2.2 Relationship Between Lactate and HF Grading

The dynamic changes in lactate concentration during HF progression serve as a direct readout of systemic metabolic and perfusion disturbances, rooted in specific cellular and molecular adaptations. In compensated HF, preserved tissue oxygenation and intact mitochondrial oxidative phosphorylation maintain lactate homeostasis. However, upon progression to decompensation, diminished cardiac output and capillary perfusion trigger a pathological shift toward anaerobic metabolism. This is driven by the stabilization of HIF-1α, which transcriptionally upregulates glycolytic enzymes and pyruvate dehydrogenase kinase, simultaneously increasing lactate production while impairing its mitochondrial oxidation and systemic clearance—a state of metabolic crisis [39,40,41,42,43]. The degree of hyperlactatemia correlates with New York Heart Association classification and is mechanistically underpinned by acquired mitochondrial defects, suppression of fatty acid oxidation via peroxisome proliferator-activated receptor alpha (PPARα)/PGC-1α downregulation, and enhanced glycolytic flux [36,41].

At the cellular level, lactate serves as a multi-functional metabolite and signaling center. Besides being involved in the Cori cycle, it triggers post-translational lysine lactylation on both histone and non-histone proteins; for example, the lactylation of histones at the promoters of fibrotic genes makes chromatin more open and promotes transcriptional activation, but the lactylation of metabolic enzymes like HADHA can directly reduce their activity, forming a feed-forward mechanism that worsens metabolic remodeling [10,42], and it also regulates the inflammatory environment by controlling the activation of the NLR family pyrin domain-containing 3 (NLRP3) inflammasome and nuclear factor kappa B (NF-κB) signaling, and aggravates oxidative stress by disturbing the mitochondrial redox equilibrium; moreover, increased intracellular lactate decreases cytosolic pH, which directly suppresses sarcoplasmic reticulum calcium ATPase activity, hinders calcium re-uptake, and consequently impairs cardiomyocyte contractility [44,45].

Clinically, continuous hyperlactatemia indicates the collapse of compensatory hemodynamic and metabolic reserves and foretells death, especially in cases of acute decompensated heart failure and cardiogenic shock [46,47], the lactate/albumin ratio (LAR) which consists of an acute indicator of glycolytic stress (lactate) and a factor showing chronic inflammation, nutrition, and liver function (albumin) offers superior risk stratification compared to each individual value separately [48,49], and methods for continuous monitoring such as subcutaneous enzymatic sensors could record lactate kinetics in real-time allowing for a dynamic evaluation of how effective treatments are and providing support for more mechanism-based management in future research.

### 2.3 Role of Lactate in Myocardial Remodeling

#### 2.3.1 Effects of Lactate on Cardiomyocytes

Lactate has come into being as a multi-functional immunometabolic signaling molecule which is of great significance in regulating inflammatory processes via various receptor-mediated and intracellular ways, it attaches to GPR81 causing a decrease in intracellular cyclic adenosine monophosphate (cAMP) levels and then suppressing the production of pro-inflammatory cytokines in immune cells like macrophages and dendritic cells [50,51], at the same time it modifies the NF-κB pathway by making the inhibitor of kappa B alpha (IκBα) stable and hindering p65 phosphorylation thus weakening the transcription of crucial inflammatory mediators including tumor necrosis factor alpha (TNF-α), interleukin 1 beta (IL-1β), and interleukin 8 (IL-8) in numerous experimental models [52,53], besides membrane receptor signaling it brings about histone lactylation especially at H3K18 changing chromatin accessibility and driving the expression of genes related to anti-inflammatory and repair processes effectively reforming transcriptional responses to metabolic signals [54,55], moreover it stops mast cell activation by repressing the Mas-related G protein-coupled receptor member X2 receptor so that degranulation and chemokine release in allergic inflammation are reduced [56], and it also affects inflammasome activity as shown by Lactobacillus paracasei which secretes lactate to increase interleukin 10 (IL-10) production and nucleotide-binding oligomerization domain 2 signaling thus inhibiting the NLRP3 inflammasome [57], these multi-level mechanisms make lactate a central metabolic immune checkpoint.

**Fig. 3.**
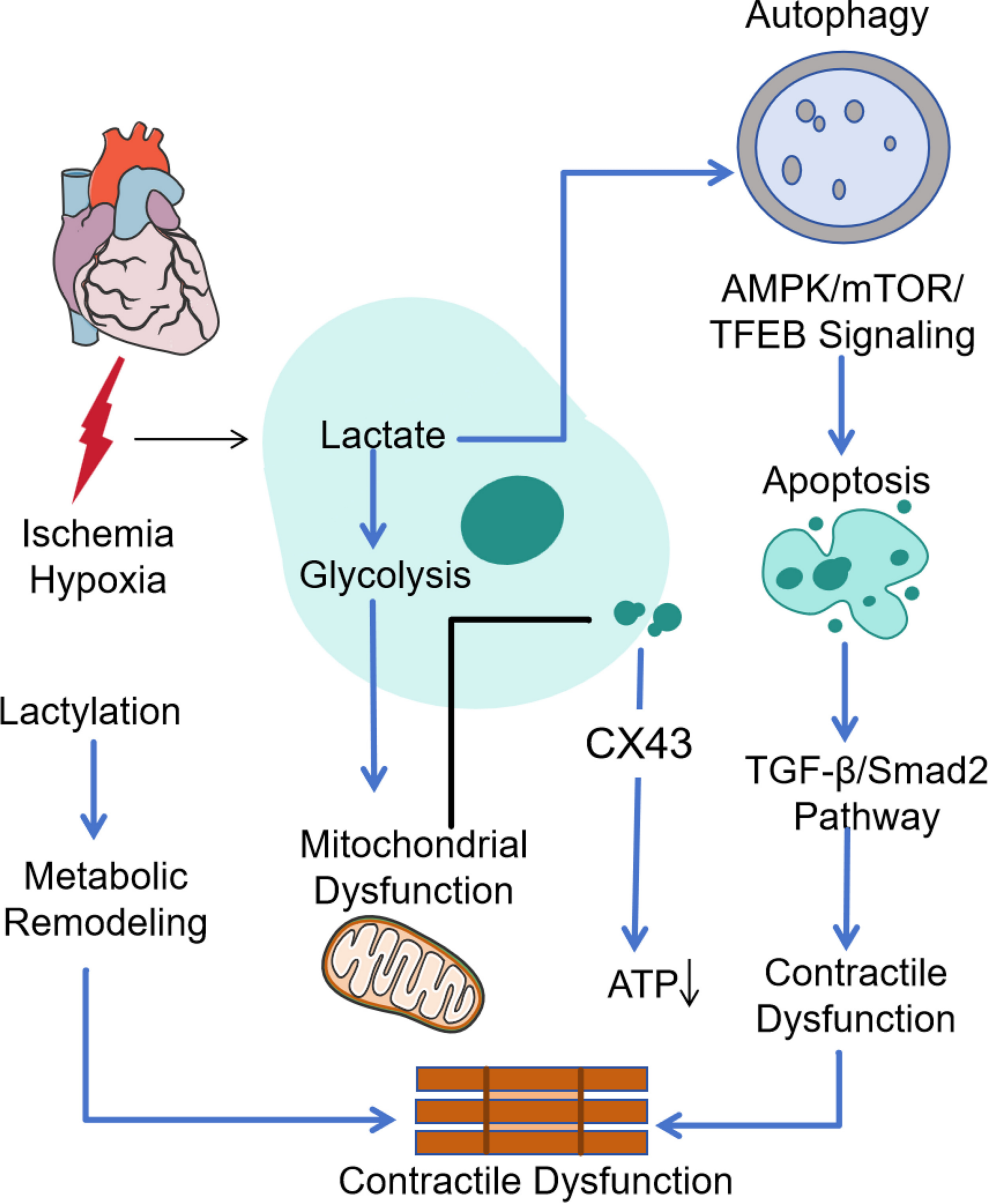
**Effects of lactate on cardiomyocytes**. CX43, connexin 43; ATP, adenosine triphosphate; AMPK, AMP-activated protein kinase; mTOR, mechanistic target of rapamycin; TFEB, transcription factor EB; TGF-β, transforming growth factor-beta. The downward arrow next to ATP denotes reduced ATP production due to mitochondrial dysfunction.

In the situation of heart failure (HF) and myocardial injury, lactate has spatially and temporally controlled impacts on cardiac immunity and greatly affects macrophage polarization by regulating metabolic-epigenetic cross-talk; when there is an ischemic condition, it makes HIF-1α more stable by inhibiting prolyl hydroxylase domain protein 2, leading to the release of IL-1β and M1-like pro-inflammatory activation [58,59,60], but during the reparative stages, it promotes M2-like polarization through the expression of arginase 1 and other anti-inflammatory genes driven by lactylation, which helps in tissue resolution and repair [61], and this dual function shows how the role of lactate in cardiac immunometabolism depends on the context; new therapeutic methods take advantage of these mechanisms as curcumin reduces inflammation partly by suppressing NF-κB via lactate, the degradation products of poly-L-lactate change macrophage metabolism to decrease foam cell formation, and the administration of probiotics with lactate-producing species systematically modulates glucose/lipid metabolism, thus influencing the cardiac inflammatory state [62,63], and importantly, lactate-mediated immunomodulation causes the suppression of key cytokines (TNF-α, interleukin 6 (IL-6), IL-1β) in the myocardium which are strongly related to the progress of HF [64,65], so understanding these complex mechanisms provides new chances for therapeutic targeting of lactate pathways to balance immune responses and enhance outcomes in HF (Results are shown in Fig. 3).

#### 2.3.2 Lactate Metabolism and Signaling Pathways: Roles in Cardiomyocytes and Fibroblasts

Lactate works through a two-fold, cell-type-specific mechanism in the heart, which is of great significance for integrating metabolic and signaling functions; in cardiomyocytes, lactate was mainly brought in through MCT1, then it was changed back to pyruvate by LDHB and after that oxidized in the mitochondria to provide energy for ATP synthesis, thus directly supporting the contractile ability, while in cardiac fibroblasts and other non-myocyte cells, lactate mainly served as a signaling molecule by activating GPR81, which set off downstream pathways that stabilized HIF-1α and activated NF-κB, leading to the promotion of pro-inflammatory and pro-fibrotic programs at the transcriptional level and driving maladaptive remodeling [66,67], and the transport of lactate via MCT1 and its sensing through GPR81 regulated the function of this molecule in the heart in terms of space, controlling both the supply of energy and the communication between cells.

**Fig. 4.**
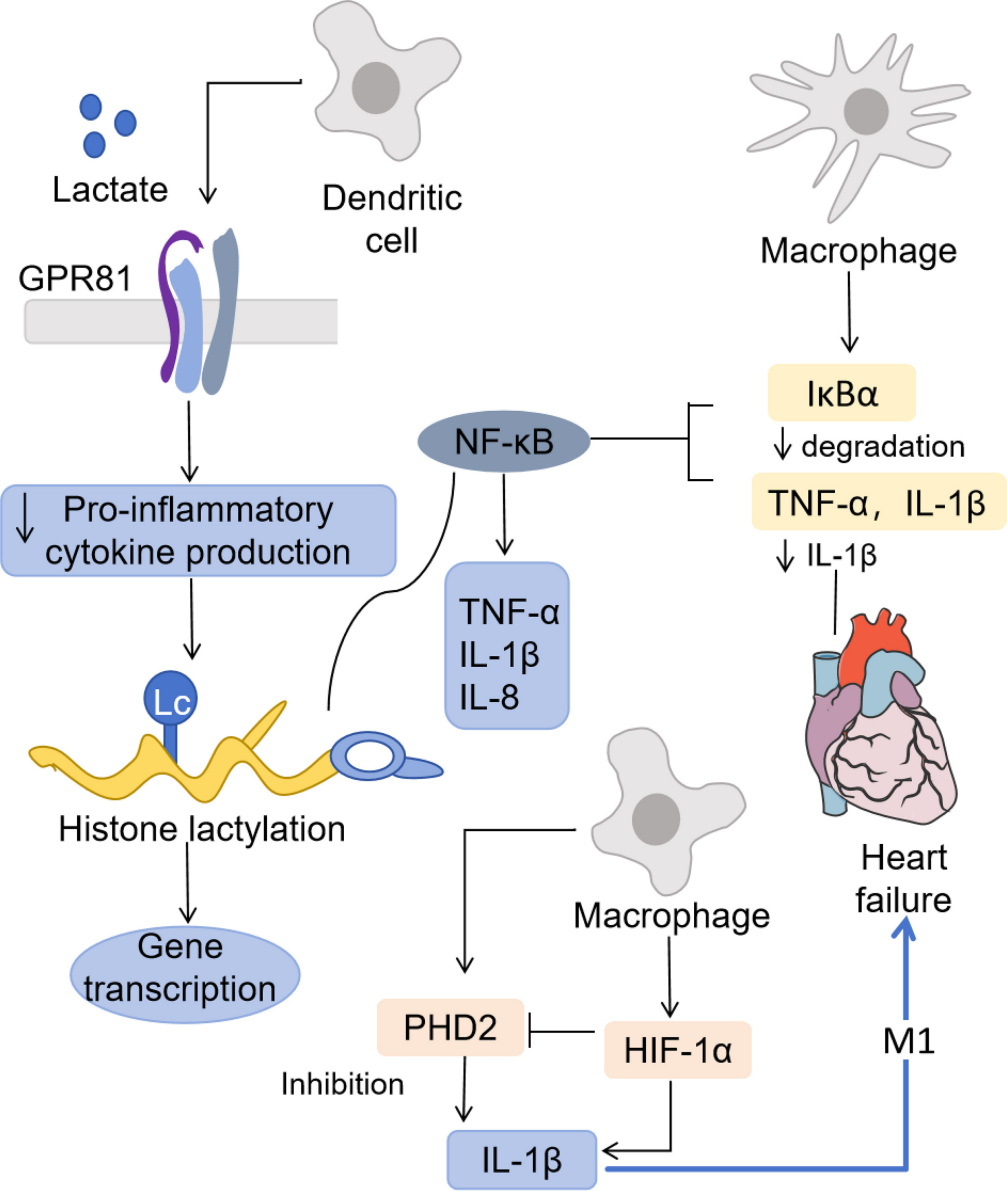
**Interaction between lactate and inflammatory responses**. NF-κB, nuclear factor kappa B; TNF-α, tumor necrosis factor alpha; IL-1β, interleukin 1 beta; IL-8, interleukin 8; IκBα, inhibitor of kappa B alpha; PHD2, prolyl hydroxylase domain-containing protein 2; HIF-1α, hypoxia inducible factor 1 alpha.

Lactate occupies such an important position right where metabolism and signaling intersect that it is a crucial point in the pathophysiology of heart failure (HF), so future therapeutic development might center on selectively regulating these different pathways, for example, promoting cardiomyocyte lactate oxidation via MCT1 or blocking the GPR81-mediated profibrotic signaling in fibroblasts, to correct bioenergetic shortages and reduce harmful structural changes at the same time, thus bringing back global cardiac balance (Results are shown in Fig. 4).

#### 2.3.3 Lactate-Induced Autophagy

Emerging evidence suggests that lactate serves not only as a metabolic substrate but also as a signaling mediator that links glycolysis with autophagy—a fundamental cellular housekeeping and stress adaptation process. For instance, under nutrient deprivation or metabolic stress, increased activity of LDHA elevates lactate production, which in turn mediates lactylation of phosphatidylinositol 3-kinase catalytic subunit type 3/vacuolar protein sorting 34 (VPS34) at key lysine residues (e.g., K356, K781). This modification enhances VPS34 lipid kinase activity and promotes autophagic flux, a mechanism demonstrated in muscle and cancer cells [68]. Thus, lactate connects two evolutionarily conserved pathways, glycolysis and autophagy, allowing cells to dynamically adjust to metabolic stress by clearing damaged components and maintaining homeostasis.

#### 2.3.4 Protein Lactylation and Mitochondrial/Energy Metabolism Regulation

Prolonged elevation of lactate levels or malfunctioning of lactate clearance (LC)/transport can result in more extensive outcomes than just affecting autophagy and in heart muscle cells (cardiomyocytes), a recent proteomic study showed that long-term or stress-related lactate buildup leads to lactylation of HADHA at residues K166 and K728 which significantly reduces its enzyme activity, thus impairing mitochondrial function, fatty-acid breakdown, and oxidative phosphorylation, lessening ATP generation and contractile strength, eventually weakening heart function [69] and these results emphasize the two-fold nature of lactate-induced lactylation as although it might boost adaptive autophagy during short-term stress, too much or prolonged lactate could be harmful by altering metabolic enzymes and disturbing energy metabolism in the heart.

### 2.4 Potential Therapeutic Targets of Lactate

#### 2.4.1 Targeted Therapy of Lactate Metabolism-Related Enzymes

Lactate dehydrogenase (LDH) is an extremely important enzyme responsible for the reversible transformation between pyruvate and lactate, and its predominant form, lactate dehydrogenase A (LDHA), plays a crucial role in promoting glycolysis and modifying the way cells metabolize energy in diseases like cancer and heart failure (HF), and newly-emerging isoforms, such as LDHAα, exhibit structurally enhanced catalytic efficacy and modified allosteric regulation, resulting in augmented glycolytic output and presenting fresh therapeutic targets for intervention [70], and the development of LDH inhibitors, ranging from natural compounds to engineered nanozymes, specifically targets these isoforms, disturbing pathological lactate accumulation via competitive or allosteric inhibition while providing better catalytic stability and tissue specificity [71,72], and in HF, high levels of serum LDH are strongly associated with mortality, indicating not only tissue hypoxia but also systemic oxidative stress and defective metabolic clearance, thus strengthening its dual function as a prognostic biomarker and a therapeutic target [73], and pharmacological substances like silybin and isoliquiritigenin reestablish cardiac bioenergetics by reducing LDHA expression and activity, consequently regulating downstream glycolytic and mitochondrial pathways, showing the translational significance of precisely adjusting lactate metabolism [19].

Therapeutic strategies that modulate lactate biology span molecular, cellular, and systemic levels. Polyphenols and hydrogen sulfide donors can reduce oxidative injury and lower LDH activity, in part by activating the Nrf2 antioxidant program and improving mitochondrial redox balance [74,75]. Exercise training reprograms myocardial metabolism in preclinical models by shifting LDH isozyme composition and increasing oxidative capacity, partly through PGC-1α upregulation [8,76]. At the epigenetic level, histone and enzyme lactylation appears to regulate gene expression and fibrotic signaling in HF [77]. Activation of the β3-adrenergic receptor supports redox homeostasis by enhancing Nrf2-driven antioxidant defense and NADPH regeneration [78]. Gene-targeting approaches (e.g., small interfering RNA (siRNA) against glycolytic enzymes) further illustrate the potential to directly modulate lactate-driven pathways and attenuate pathological fibrosis [79]. Together, these pharmacologic, nutraceutical, and lifestyle approaches aim to restore energetic flexibility and slow adverse remodeling, but future work should define when lactate is adaptive versus harmful in HF.

#### 2.4.2 Development of Novel Lactate-Related Drugs

The development of new-generation drug therapies for heart failure (HF) has been more and more focused on precisely regulating lactate metabolism and its connected signaling systems, making use of lactate’s two-fold role as both an energy source and a controller of cell signaling, for example, trimetazidine improves the heart muscle’s metabolic effectiveness by changing how substrates are utilized so that it favors lactate oxidation via activating AMP-activated protein kinase (AMPK) allosterically and increasing the level of PPARα at the transcriptional stage, thus bringing back ATP production when there is insufficient blood flow, additionally, besides dealing with energy matters, new epigenetic methods have come into being such as inhibiting *LDHA* reduces histone lactylation which was only recently found out as a post-translational modification causing changes in the expression of genes related to enlargement of the heart and stopping the harmful remodeling of the heart, all of these methods aimed to restore metabolic adaptability and slow down the structural damage in heart failure.

#### 2.4.3 Mechanistic Model: Lactate-Shuttle Efficiency in Cardiac Metabolism and HF

We propose an integrated “lactate-shuttle efficiency” model to reconcile the context-dependent dual roles of lactate in the stressed or failing heart. In this framework, the net impact of lactate, as either a beneficial metabolic substrate or a detrimental signaling molecule, is determined by the dynamic balance among its production, transport efficiency (primarily via MCT1/4), mitochondrial clearance and oxidation capacity, and the prevailing metabolic and redox state of cardiac cells. Under conditions of elevated energy demand, ischemia, or impaired fatty acid oxidation, lactate produced by glycolytic cells can be shuttled through intra- or intercellular lactate transport into cardiomyocytes and oxidized to support ATP generation, thereby preserving cardiac function and representing its protective, fuel-providing role [6,67,80].

On the contrary, when lactate generation exceeds the body’s capacity to clear it—because of decreased activity of transporters, malfunctioning mitochondria, or imbalances in oxidation—lactate builds up, causing acidification both inside and outside cells, creating metabolic strain, triggering abnormal tissue changes, and eventually resulting in a decline in function, showing its harmful part in the progress of heart failure [7,81].

**Fig. 5.**
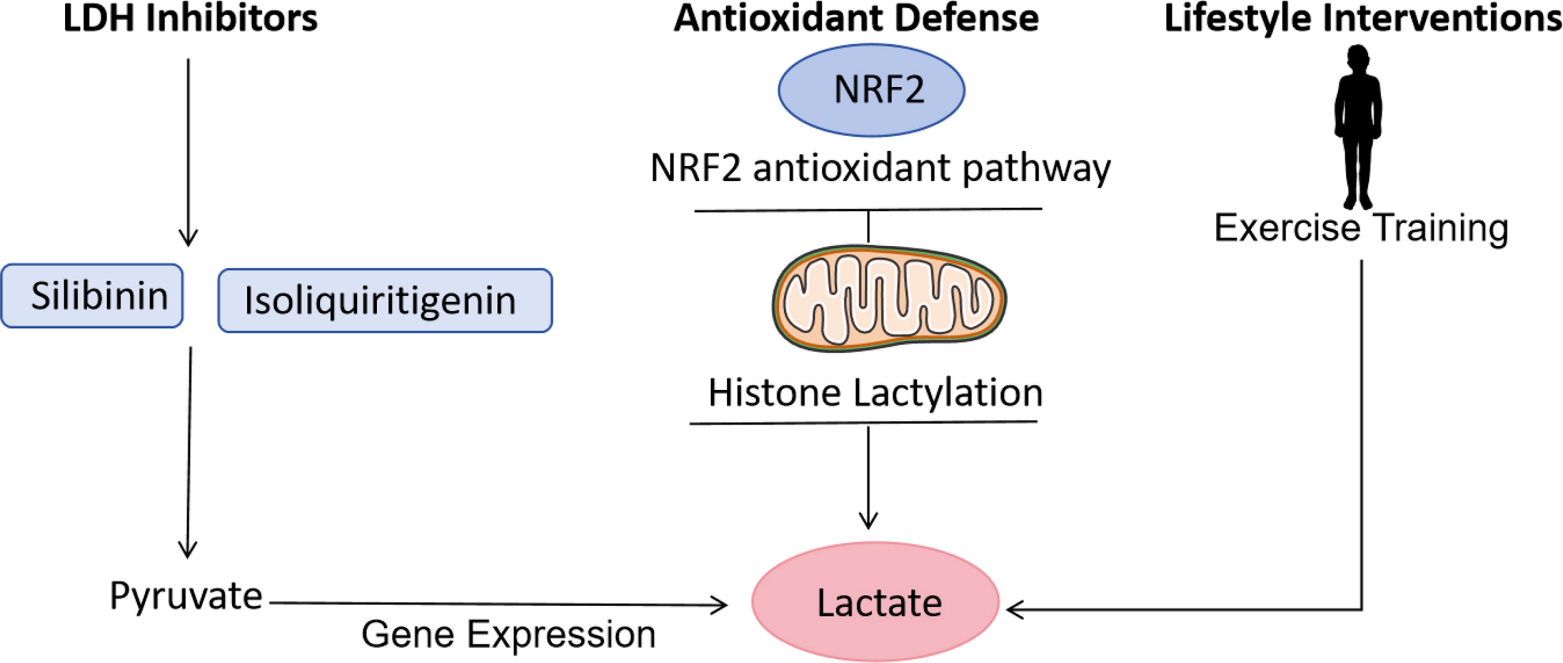
**Potential therapeutic targets of lactate**. NRF2, nuclear factor erythroid 2-related factor 2.

This model presents a mechanistic foundation for the dual-effect phenomena of lactate within cardiac pathology and also offers a guiding principle for future research; we recommended that studies combine isotope-based metabolic flux analysis, genetic or pharmacological interference with lactate transporters, long-term lactate exposure models, and multi-dimensional functional phenotyping so as to determine crucial thresholds regarding concentration, exposure length, and redox environment which determined whether lactate was advantageous (“friend”) or detrimental (“foe”) in heart failure (Results are shown in Fig. 5).

### 2.5 Clinical Application Prospects of Lactate

#### 2.5.1 Standardization of Lactate as a Biomarker

Lactate is widely used as a biomarker in HF and other clinical settings. Because it reflects tissue hypoxia, metabolic stress, and disease severity, standardized monitoring protocols are needed to ensure that measurements are comparable and clinically interpretable. Current practice varies by sampling method, specimen type, assay platform, and timing. Blood lactate remains the reference standard, while noninvasive approaches (e.g., sweat or saliva lactate) are being explored but still face challenges in bioequivalence and reproducibility [82,83]. Interpretation is further complicated by patient factors (demographics, comorbidities) and by clinical interventions. Intensive care studies also show that lactate testing frequency can vary with patient characteristics and access to care, supporting the need for more consistent monitoring [84]. Point-of-care biosensors with rapid readouts are promising for real-time monitoring, but they require careful validation and calibration against standardized blood measurements [85,86]. Overall, consensus on sampling, assays, and interpretive criteria is essential to fully realize lactate’s value as a biomarker across HF phenotypes and care settings.

Several elements restrict the day-to-day utilization of lactate as a biomarker for making decisions such as methodological distinctions, biological fluctuation, and the failure to be fully incorporated into clinical procedures; one substantial shortcoming was the absence of universally agreed-upon cut-offs which could classify the severity of diseases or foresee results among diverse heart failure populations; non-invasive surveillance (through sweat or saliva) might have made continuous evaluation possible but linking these readings to blood lactate remained challenging due to bioequivalence problems [82,83]; machine learning techniques could enhance lactate-based risk classification by integrating lactate dynamics with other biomarkers and clinical data and dealing with assay variations, establishing clear thresholds, and better integrating into clinical practice would have strengthened the influence of lactate monitoring on patient outcomes.

#### 2.5.2 Role of Lactate in Personalized Therapy

##### Lactate Dynamic Monitoring in Personalized Therapy

Lactate has turned into a crucial biomarker in the handling of heart failure (HF), especially because of its role in signaling tissue hypoperfusion and metabolic strain, and the dynamic monitoring of lactate levels offers precious information about a patient’s hemodynamic state and their reaction to treatments, enabling a more individualized treatment plan; in acute heart failure (AHF) and advanced HF, high lactate levels often point to inadequate oxygen supply and enhanced anaerobic metabolism, which are linked to worse clinical results, for instance, in cardiogenic shock (CS), repeated lactate evaluations and lactate clearance (LC) were shown to predict survival and guide the intensity of treatment, as higher lactate levels and poor clearance corresponded to a greater death risk [15], and this dynamic assessment is better than static lactate measurements as it grasps the progress of metabolic recovery or deterioration, thus giving real-time views on how effective interventions like inotropic support, mechanical circulatory aid, or fluid resuscitation are.

In the situation of heart transplantation, post-operative hyperlactatemia was often seen and indicated the complex metabolic and hemodynamic disorders after surgery; continuous lactate surveillance in heart transplant receivers showed diverse lactate trends and some patients had constant hyperlactatemia which could suggest graft malfunction or systemic problems [87]; recognizing these patterns allowed clinicians to evaluate the risk and adapt post-operative management approaches which might involve changes in immunosuppression, hemodynamic support, and infection observation.

Furthermore, lactate surveillance was effectively combined with other biomarkers and clinical indicators to improve prognostic models; for example, in patients with sepsis and heart failure (HF), the integration of lactate levels with clinical scoring systems enhanced the prediction of short-term survival and helped in formulating individualized treatment methods [88].

Point-of-care testing and continuous monitoring devices have facilitated the incorporation of lactate trends into bedside care and these tools could enable timely interventions by making it possible to adjust treatment according to lactate trajectories instead of relying on single measurements, but lactate thresholds and monitoring intervals still needed standardization across heart failure (HF) phenotypes and clinical settings.

### 2.6 Future Research Directions

#### 2.6.1 Lactate Metabolism and Basic Research in HF

Lactate is no longer regarded merely as a by-product of glycolysis but rather as a regulator of metabolic reorganization in heart failure (HF), and in the failing heart, an elevated glycolytic flow elevates lactate levels which then functions as both an oxidizable fuel and a signaling entity, partly via lysine lactylation (Kla), while delactylases like sirtuin 1 (SIRT1), SIRT3, and histone deacetylase 3 strip away lactyl groups in reaction to metabolic signals (for instance, the NAD^+^/NADH ratio), underpinning lactylation as a responsive metabolic signal [29,89], and stress-induced enhancement of LDHA induces cardiomyocyte enlargement, on the other hand, LDHA deficiency might lead to heart malfunction and in certain models, lactate supplementation restores this trait through stabilizing N-myc downstream-regulated gene 3 (NDRG3) and activating extracellular signal-regulated kinase signaling [87], also lactate can alter cardiac structural proteins such as the lactylation of α-myosin heavy chain at lysine 1897 being associated with sarcomere stability and contractility and the absence of this modification corresponding to the progress of HF [88], nevertheless, despite these developments, definite *in vivo* proof linking site-specific lactylation in cardiac cells to results like contractility or fibrosis remains scarce [90] so future research should employ genetic means (for example, lactylation-defective knock-in mutants) and cell-specific adjustment of lactylation/delactylation enzymes to determine causation, and long-term lactate exposure could hamper mitochondrial fatty acid oxidation, boost reactive oxygen species (ROS), and decrease respiratory capacity, contributing to metabolic rigidity [91], moreover, transporters like MPC and MCT4 govern the pyruvate-lactate equilibrium, further emphasizing lactate’s function in coordinating metabolic and structural reorganization in HF [10].

**Fig. 6.**
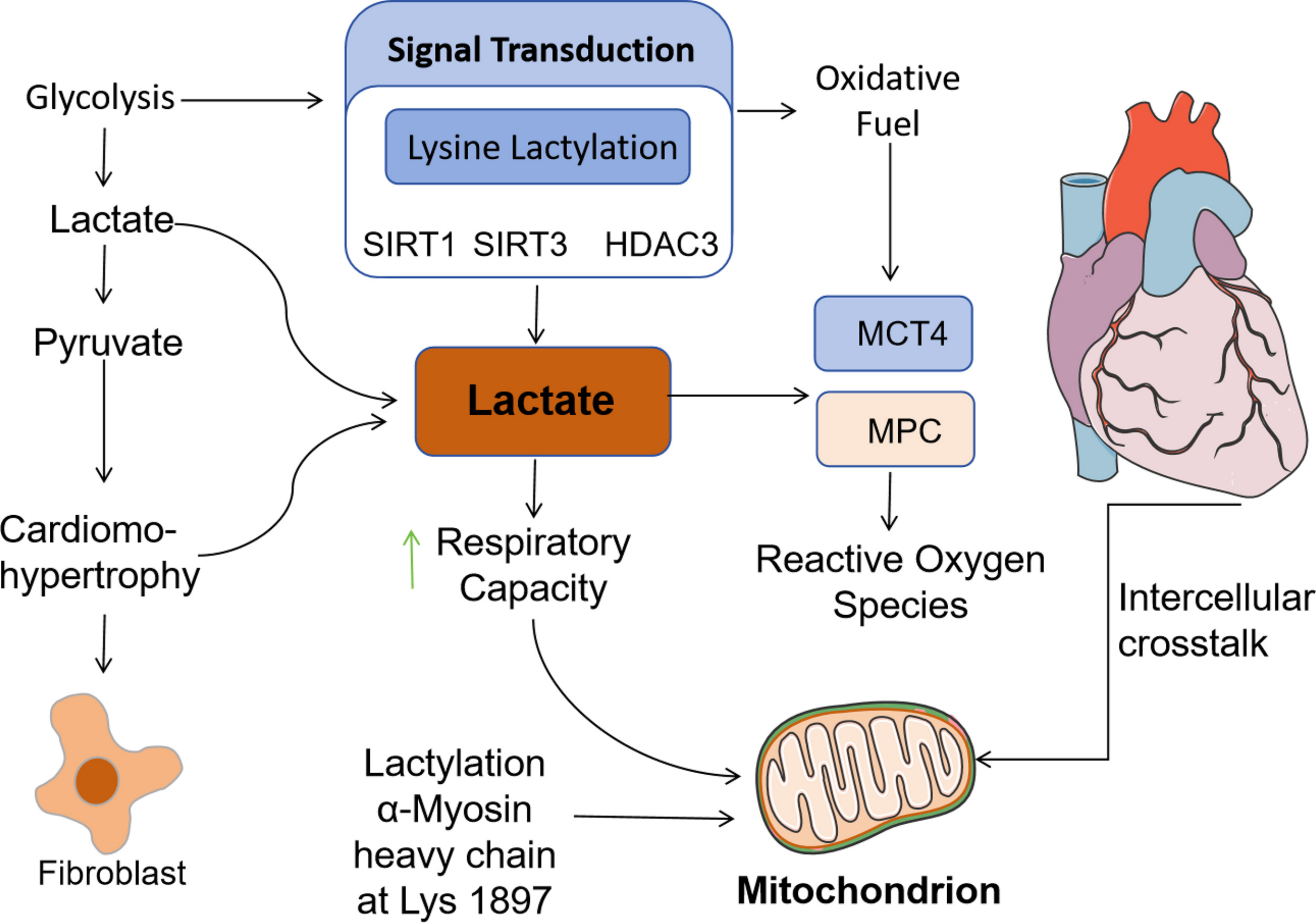
**Lactate metabolism and basic research in heart failure**. HDAC3, histone deacetylase 3; MCT4, monocarboxylate transporter 4; MPC, mitochondrial pyruvate carrier.

Besides the effects within cardiomyocytes, lactate is involved in cross-talk between cells and at the pathway level which forms heart failure (HF), and the pyruvate-lactate axis plays a crucial role in maintaining the energy and redox balance in the heart so when it was dysregulated, the contractile function might be damaged but treatments like Fuzi decoction could assist partially by re-establishing this equilibrium [92], and lactate also connects glucose and fatty acid metabolism for instance empagliflozin decreased glycolytic lactate production while increasing the synthesis of α-ketoglutarate from fatty acids enhancing metabolic adaptability in diabetic cardiomyopathy [93], and the “cardiac lactate shuttle” model further illustrates the metabolic connection where lactate produced by fibroblasts is utilized by cardiomyocytes during oxidation aiding in adaptation under stress or aging [67], and collectively the flow of substrates, transportation between cells, and regulated lactylation position lactate right in the middle of the pathophysiology of HF, and determining the *in vivo* influence of site-specific protein lactylation would be significant for turning these discoveries into targeted metabolic therapies (Results are shown in Fig. 6).

#### 2.6.2 Clinical Research Deepening and Promotion

Translating lactate-focused mechanistic findings into HF therapies will require clinical trials designed around the syndrome’s multifactorial biology. Endpoints should include arterial lactate kinetics, hemodynamic measures, and molecular readouts such as lactylation of metabolic enzymes (e.g., acetyl-coenzyme A acyltransferase 2) to link efficacy to mechanism [94,95]. Because lactate alone may not capture impaired cardiac output or regional perfusion, invasive hemodynamic monitoring remains important in many settings [37].

A number of therapeutic methods might be beneficial for heart failure (HF) through the modulation of lactate-associated pathways, for example, Levosimendan was able to enhance tissue perfusion and assist in lactate clearance within acute heart failure (AHF) by strengthening contractility and inducing peripheral vasodilation [42], nanocarrier systems like exosomes loaded with curcumin could allow for targeted delivery to cardiomyocytes which could suppress LDH activity and lessen oxidative damage [96], combined metabolic and immunomodulatory approaches such as β1-selective antagonists in septic shock were likely to restrict both catecholamine-induced lactate generation and inflammatory activation at the same time [97], eventually, predictive models that integrated dynamic lactate levels with validated clinical scores for instance the Sequential Organ Failure Assessment and the Acute Physiology and Chronic Health Evaluation II could improve risk classification and direct personalized treatment thus promoting a shift towards precise management in HF care targeting lactate [40].

#### 2.6.3 Biological Rationale and Clinical Standardization for LAR

LAR serves as an integrated prognostic indicator in heart failure as it grasps both acute metabolic strain and long-term inflammatory-metabolic reserve [98], increased lactate indicates anaerobic metabolism spurred by hypoperfusion and mitochondrial malfunction while decreased albumin shows continuous inflammation, malnutrition, and liver synthetic impairment, these alterations collectively diminish antioxidant ability and metabolic adaptability, and through integrating these interconnected yet separate aspects, LAR was able to enhance risk classification more effectively than using lactate alone.

LAR presents a combined result of two interconnected but separate pathological aspects: real-time glycolytic flow and energy emergency (lactate), as well as long-term immunometabolic capacity and tissue wholeness (albumin), and its better predictive ability compared to just lactate comes from this double representation, which allows for more precise grouping of patients who are more susceptible to metabolic-inflammatory issues.

For clinical adoption to be supported, having standardized protocols for assessing LAR is of great significance, these protocols need to cover the synchronized measurement of lactate and albumin from the very same blood sample, if possible, within the initial hour after admission, by using either arterial or venous sources consistently so as to reduce pre-analytical variability, setting up LAR thresholds specific to different phenotypes, such as for those with ischemic conditions, diabetes, or heart failure in the elderly, would enhance the accuracy of predicting 30-day mortality and the necessity for rehospitalization, moreover, repeated LAR assessments could monitor how patients respond to decongestive or anti-inflammatory treatments, showing whether hypoperfusion and systemic inflammation have been resolved and consequently guiding the adjustment of treatment dynamically.

Widespread implementation of standardized LAR measurement may significantly enhance risk assessment and enable more personalized management strategies in HF.

#### 2.6.4 Strategies to Enhance Cardiac Specificity in Targeted Interventions

Targeting key elements within central metabolism like *LDHA* and MCTs is a hopeful but difficult therapeutic approach for heart failure (HF), considering their crucial functions in lactate metabolism and the energy supply of the heart, nevertheless, the widespread presence of these targets across the heart, skeletal muscle, and central nervous system brings up worries about side-effects when they are inhibited throughout the body, so to deal with this, new ways of delivering treatment specifically to certain tissues have been created, for example, modifying exosomes or artificial nanoparticles with peptides that guide them to the heart (such as those that bind to specific markers on heart muscle cells or the blood vessels surrounding them) makes it easier for them to be taken up by receptors mainly in the heart, these systems take advantage of differences in how cells absorb substances and in the permeability of blood vessels, greatly increasing the amount in the heart while reducing the amount in the skeletal muscle and the central nervous system, research shows that exosomes with these guiding molecules can strongly target the heart without being trapped in large amounts in the liver or spleen.

Besides delivery vectors, molecular precision could be achieved via allosteric inhibitors or isoform-selective drug designs which take advantage of the structural differences between the cardiac and peripheral forms of *LDHA* or MCTs, for instance, designing compounds to interact with areas specific to the heart’s regulation or sites modified after translation might reduce interference with glycolysis in skeletal muscles and metabolism in neurons.

Moreover, combining gene therapy approaches like adeno-associated virus vectors together with cardiac-specific promoters (for instance, troponin T or myosin heavy chain 6) makes it possible for therapeutic siRNAs, microRNA, or mRNA constructs to be expressed in a spatially restricted manner and this sort of transcriptional targeting takes advantage of cell-type-specific enhancer-promoter activities which restricts transgene expression mainly to cardiomyocytes thus avoiding systemic side effects.

These approaches which cover biomaterial engineering, structural pharmacology, and transcriptional targeting can be utilized together and provide feasible means to enhance cardiac specificity while targeting lactate-related pathways; future research was supposed to verify the on-target cardiac impacts and measure the extracardiac outcomes by employing multimodal imaging, single-cell transcriptomics, and metabolomic flux analyses so as to speed up the process of translating findings into treatments for heart failure (HF) therapy.

#### 2.6.5 Decoding the Gut–Cardiac Lactate Axis in HF

Recent research regarding the gut-heart connection has revealed a fascinating association between an imbalanced gut microbiota and the progression of heart failure (HF), which is mainly mediated by metabolites from the microbiota that control systemic inflammation, the stability of blood vessels, and the metabolic balance of the host [99], and the gut microbiota is also an unconventional source of systemic lactate, producing both L- and D-forms, which enters the bloodstream and might impact distant organs like the heart [100], so considering the well-known functions of lactate in the energy supply of heart muscle cells, the transfer of metabolites between cells, and the regulation of signaling pathways such as the activation of HIF-1α and the NLRP3 inflammasome, lactate from the gut may considerably add to the lactate level in the heart, and this external flow of lactate could modify the redox status, affect mitochondrial operation, and encourage lactylation-related epigenetic changes in heart muscle cells, thus speeding up metabolic adjustment and malfunction in HF.

To mechanistically analyze this gut-cardiac lactate connection, future research ought to utilize multi-omics methods by combining metagenomics, metabolomics (specifically lactate isomer analysis), and transcriptomics while also evaluating gut barrier stability (for instance, zonulin and lipopolysaccharide levels) in HF groups with known traits, and additional animal models like mice lacking microbiota but inoculated with bacteria that produce or consume lactate might be useful in determining cause and effect and clarifying how gut-derived lactate influences heart enlargement, scarring, and energy utilization, and if these mechanisms were understood, new ways to target the microbiome—such as prebiotics, probiotics, or substances that prevent bacteria from making lactate—could be found to regulate lactate movement and enhance results in HF.

## 3. Conclusion

In general, there is an increasing amount of evidence indicating that lactate has various functions in heart failure (HF), rather than merely being a metabolic byproduct or a sign of disease severity, as was discussed earlier, it could impact myocardial remolding, inflammatory signaling, and energy metabolism, so this more comprehensive perspective made lactate not just an indication of HF severity but also possibly a factor driving disease progress and a potential target for therapy.

Making sense of published findings is still quite difficult as numerous studies depict routes by which lactate impacts heart cells and the overall inflammatory state, thus reinforcing the idea of focusing on lactate metabolism in heart failure (HF), but meanwhile, outcomes could vary concerning particular molecular processes, effects depending on the stage, and interactions with other substances and signaling pathways, so to deal with these matters, combined metabolic, molecular, and clinical data were required in order to understand lactate’s part in the broader picture of HF pathophysiology.

The translational influence of lactate-focused interventions is also contingent upon standardized approaches for lactate measurement and metabolic profiling within clinical practice, and disparities in assays, sampling, and interpretive standards restrict the comparability among studies and complicate clinical utilization, so future research ought to set up standardized procedures that can measure lactate dynamics and associate them with clinical results, which would assist in identifying patients who are most probable to derive benefits and back well-designed clinical trials.

Importantly, therapy isn’t just about reducing lactate levels; methods that rectify aberrant lactate generation, transportation, or utilization might enhance remodeling and cut down on maladaptive inflammation, which could be beneficial to heart function and prognosis, and since lactate can act as both an energy source and a signaling molecule, interventions need to be adjusted to maintain energy support while preventing unwanted consequences like energy depletion or intensifying inflammatory pathways.

When considered as a whole, integrating the metabolism of lactate into the pathogenesis of heart failure (HF) enhances the comprehension of mechanisms and underlines novel treatment approaches, so future research needs to clarify the molecular mechanisms, confirm lactate-based biomarkers, and assess lactate-targeted interventions in strict clinical trials, and these endeavors would be expected to assist in transforming lactate biology into enhanced diagnosis, prognosis, and treatment for HF.
